# Mandibular Full-Arch Rehabilitation over 3 Straight Immediately Loaded Implants: 8 Years of Follow-Up

**DOI:** 10.1155/2019/4648959

**Published:** 2019-08-28

**Authors:** Márcio de Carvalho Formiga, Magda Nagasawa, Jamil Awad Shibli

**Affiliations:** Department of Periodontology and Oral Implantology, Dental Research Division, University of Guarulhos, Sao Paulo, Brazil

## Abstract

Mandibular full-arch restoration is a good and successful treatment option for totally edentulous patients. In the past years, several studies have described the placement of 4 to 6 implants to restore this type of case; however, an option using 3 dental implants placed in strategic and specific positions could also be an alternative. Therefore, this case report describes a full-arch rehabilitation on 3 straight, immediately loaded implants after 8 years of follow-up. The restoration presented no biological or technical complications during this follow-up period, showing that an adequate treatment plan was able to allow good results using this treatment option.

## 1. Introduction

Full-arch implant-supported fixed restoration is a very reliable option for completely edentulous patients. According to the original Bränemark protocol, four to six implants should be inserted in the interforaminal area to support a fixed, screw-retained restoration using an immediate or delayed loading protocol [1, 2]. A complementary treatment option for totally edentulous subjects is the All-on-4 concept. In this technique, two anterior implants are placed in parallel position and two distally tilted implants are placed in the most distal position, between molar and premolar areas [3]. With this configuration, the distal cantilever length can be reduced, decreasing peri-implant bone stress and consequently bone loss [4].

There are several clinical and *in vitro* studies that have concluded that both situations have similar stress distribution on the surrounding bone of the distal implants, leaving the choice of choosing one or other depending on the preference of the operator and anatomical situation [5, 6]. Early studies have proposed that in specific and well-indicated cases, full-arch rehabilitation on 3 straight immediate-loaded implants should be performed [7]. This configuration could simplify the treatment and reduce costs to the patients, in spite of the scarcity of studies with long-term follow-up periods [8, 9].

Therefore, the aim of this case report was to present the case of a patient, submitted to a mandibular fixed rehabilitation on 3 straight immediate-loaded implants, with a follow-up period of 8 years.

## 2. Case Report

**Figure 1:**
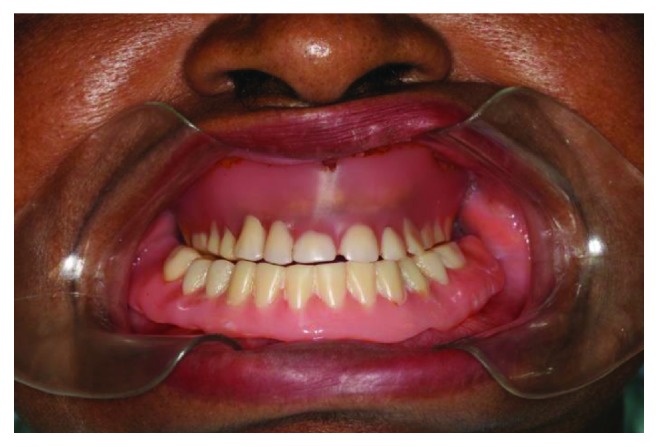
Clinical aspect of the removable complete dentures: note the absence of occlusal contact and marginal adaptation to the jaws.

**Figure 2:**
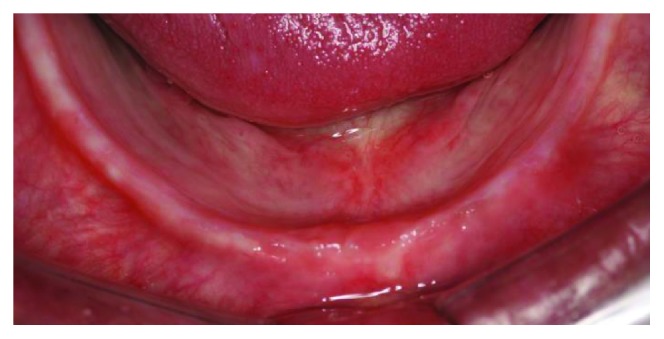
Preoperative intraoral clinical view.

**Figure 3:**
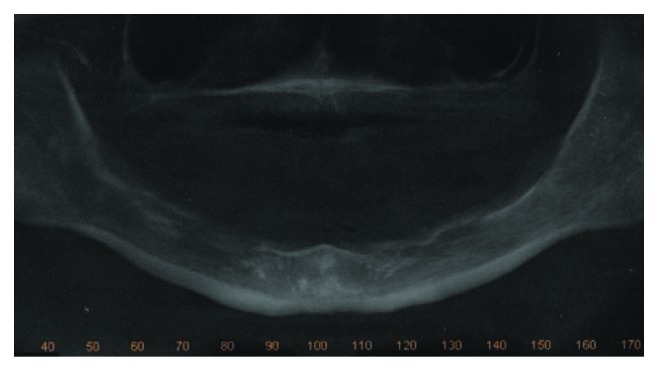
Preoperative panoramic radiograph.

The patient, a 65-year-old woman, came to our private practice in 2010, with the complaint that she could not eat without discomfort in the mental foramen region due to the use of a mandibular complete denture. The patient had been using the same dentures for 30 years (Figures 1 and 2). On clinical examination, it was possible to feel the alveolar nerve on the crest of the mandible, and its compression during clenching usually caused the patient to feel pain. After imaging analysis (Figure 3), a fixed full-arch rehabilitation on 3 straight immediately loaded implants was planned because the interforaminal distance limited the placement of four implants, according to the surgeon's experience.

**Figure 4:**
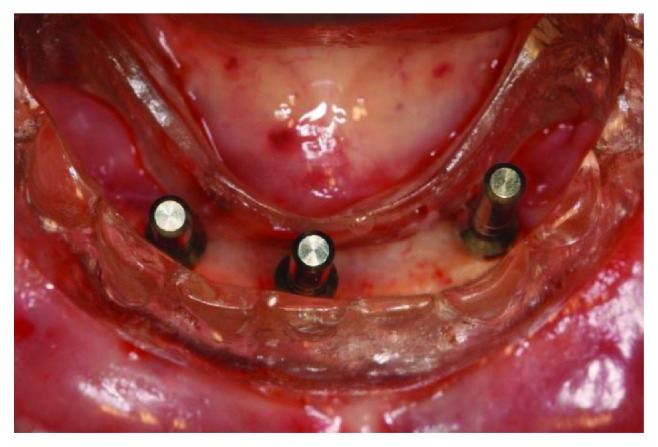
Intraoperative intraoral view of the surgical guide showing parallelism pins.

**Figure 5:**
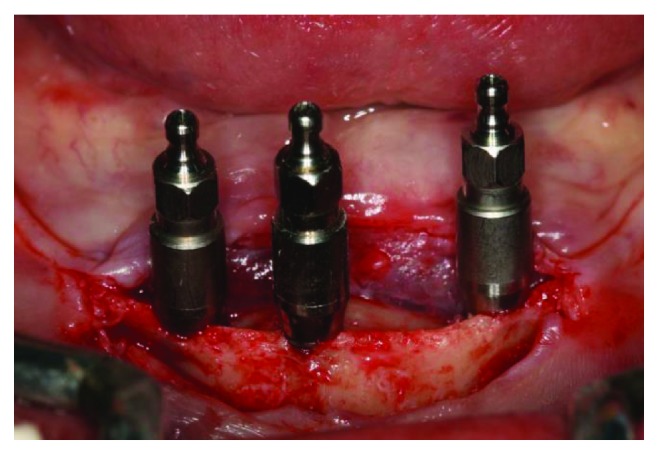
Intraoperative intraoral view of the 3 implants inserted in the jaw.

**Figure 6:**
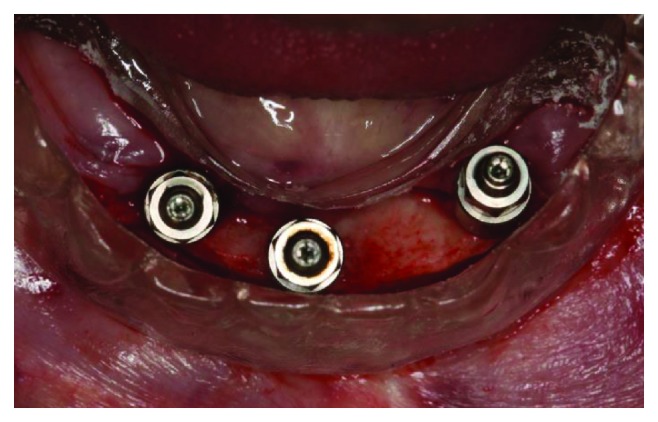
Intraoperative intraoral view of the surgical guide showing good positioning of the 3 implants.

Before the surgery, a new complete denture had to be made, with better and adequate vertical dimension, centric relation, harmonious tooth positioning, and lip support. With these parameters tested and approved by the patient and the operator, the surgical procedures were planned, using a multifunctional surgical guide to determine the best implant placement positions. After anesthesia with Articaine 4% 1 : 100,000 (DFL, Rio de Janeiro, Brazil), a crestal incision was made next to the emergence of the alveolar nerve, which we could feel by touch, to avoid any nerve damage caused by the scalpel. A full thickness incision flap was performed to expose the alveolar ridge; then, with the help of the surgical guide, the implant osteotomies were performed. The implant positions were tested with the parallel pins and the surgical guide before completing the osteotomies (Figure 4). Three Titamax GT 3.75 × 11 mm implants (Neodent, Curitiba, Brazil) were inserted in the interforaminal region (Figures 5 and 6), with an insertion torque ranging between 60 and 80 N/cm.

**Figure 7:**
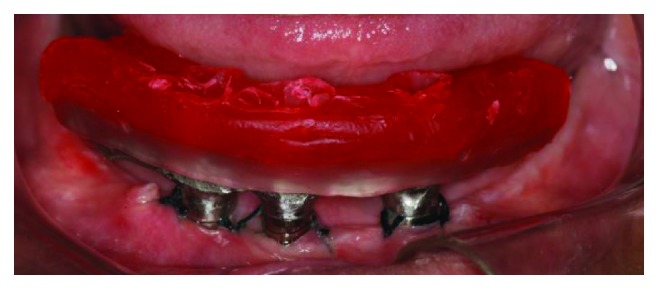
Intraoral view of the bar proof with occlusion vertical dimension wax block registration.

**Figure 8:**
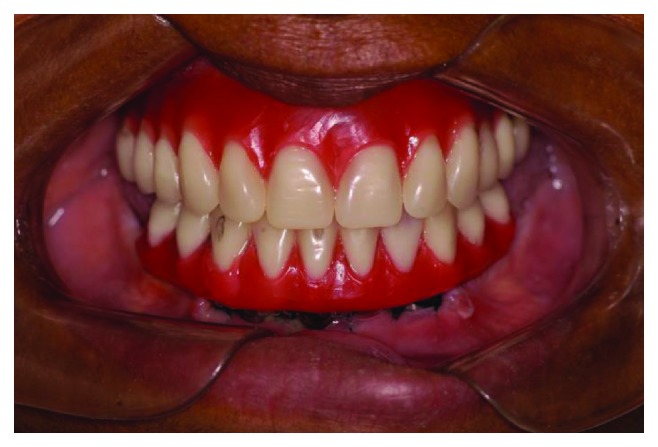
Intraoral view of the wax mounted teeth try-in.

**Figure 9:**
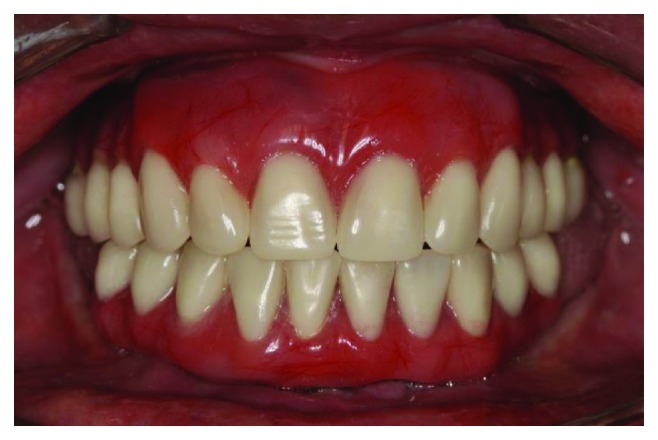
Clinical aspects of the new maxillary denture and mandibular fixed prosthetic restoration on 3 implants on the delivery day.

**Figure 10:**
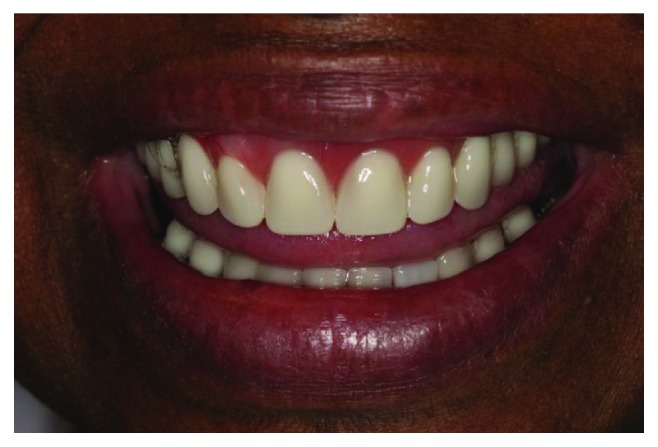
Extraoral view of the patient with the new oral rehabilitation.

The multifunctional surgical guide was used to help transfer the implant positions and register the vertical dimension and positions of the teeth. The day after surgery, the nickel-chromium metal bar, which had been made according to the position in the wax-up of the future teeth, was tried and sent for mounting the teeth, after being approved by the patient (Figure 7). On the next day, the esthetic appearance, which provided adequate lip support, smile line, positioning of the teeth, and occlusal guidelines (Figure 8), was approved. After patient and dentist approval, the prostheses were sent for full laboratory processing with acrylic. Finally, on the third day after surgery, the screw-retained full-arch rehabilitation on the three implants was installed (Figures 9 and 10).

**Figure 11:**
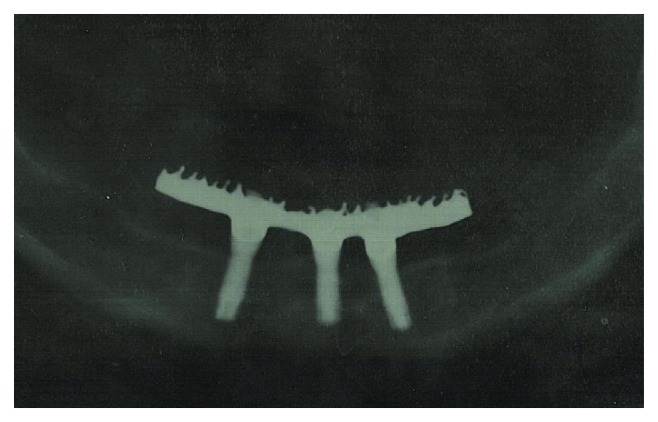
One-year postoperative follow-up panoramic radiograph.

**Figure 12:**
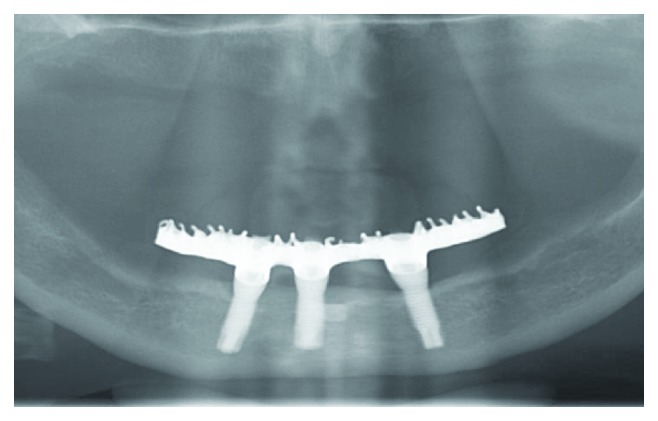
Eight-year follow-up panoramic radiograph: note that there is no bone loss around the implants.

The patient was instructed not to sleep with the opposing complete denture for 7 days, eat only soft foods, put ice bags on the surgical area for 48 hours, and take analgesic medication if necessary. The sutures were removed after seven days. The patient was recommended to return after 3, 6, and 12 months. The patient returned after one year for a clinical and radiographic evaluation (Figure 11). After this, the patient returned seven years later, for the second follow-up. This clinical evaluation showed that the fixed restoration, screw-retained on 3 implants, did not show any type of failure in the teeth or the acrylic denture base. There was no bleeding on probing (2 mm probing depths around all implants), the appearance of the gingival tissue around the implants was very good, and there were no complaints about the fixed rehabilitation. In the evaluation by panoramic radiograph, there were no aspects of bone loss or bone remodeling around the implants (Figure 12). Only a few adjustments on the complete dentures of the opposite arch were made to address minor discomfort and mucosal injuries.

## 3. Discussion

Although the full-arch fixed rehabilitation on 3 implants is not a new alternative for the treatment of completely edentulous patients [6], there are very few articles about it in the literature and even fewer articles with follow-up periods of over 3 years [9, 10]. Because of the reduced costs of this treatment modality and the simpler surgical procedure for the placement of only 3 implants for a fixed rehabilitation, the authors consider that there should be more studies about it. Furthermore, these studies should have longer follow-up periods to prove whether or not this treatment is effective or not so that it could be offered as an equivalent treatment alternative, especially for those who cannot afford the regular implant treatment options. The case presented here, with anatomical limitations for the placement of 4 implants, was taken from the files of a private practice and agreed with the conditions for the placement of 3 straight implants and an immediately loaded full-arch fixed rehabilitation.

The patient had a complete denture on the opposite arch, which could help with the success of the treatment [8]; however, in the literature, there are studies in which both arches were rehabilitated with All-on-4 or other treatment modalities, even with zirconia prostheses [10]. Immediate loading was the preference for this case, since there were no systemic or local contraindications, and the follow-up visits showed a 100% success rate, for the prosthetic components and the implants. There are studies with similar results for rehabilitation on 3 implants but with delayed load [10] and others with survival rates similar to those of the All-on-4 immediate load concept [8, 9]—a more than well documented treatment alternative for patients with an edentulous mandible [3, 10]. The implants used in this case were Neodent GT, which are one-stage single-body implants, with the only indication for use being in immediate loading mandibular protocols. Therefore, the microgap would remain above the bone crest [11], decreasing the possibility of bone remodeling around the implant neck usually seen in external and internal hexagon implants [12]. This was evident in the 1- and 8-year control panoramic radiographs of the patient, in which almost no bone loss could be seen. The result of this particular case is in agreement with one cited in a recent systematic review that assessed complications in the All-on-4 protocol, with tilted or not tilted implants [13]. The implant configuration used in this case differed from the one proposed by Costa et al. [14], but during the 8 years of follow-up, there were no failures or complications related to the implants or the metal-acrylic resin complete fixed prostheses, as reported by Bozini et al. [15]. In addition, the patient reported greater confidence in relating to other people in public because she no longer experienced discomfort while chewing, and her appearance had improved. These long-term results indicated that this could be a good option for improving the quality of life of patients who had lost their teeth, irrespective of the cause [16].

## 4. Conclusion

Within the limitations of this report, this type of rehabilitation with 3 implants in immediate function seems to be a feasible treatment option in the long term for patients with a completely edentulous mandible, when the anatomy is unfavorable or the patients cannot afford the conventional and more well-documented treatment options.
